# Behavioral responses to baited enclosure method that activates habituation in stray dogs

**DOI:** 10.14202/vetworld.2023.12-17

**Published:** 2023-01-04

**Authors:** Sumpun Thammacharoen, Sapon Semsirmboon, Visara Chit-Opas, Phak-Anong Tangcharoensin, Kran Nilkachatarn, Narongsak Chaiyabutr

**Affiliations:** 1 Department of Physiology, Faculty of Veterinary Science, Chulalongkorn University, Pathumwan, Bangkok 10330, Thailand; 2 The Academy of Science, The Royal Society of Thailand, Dusit, Bangkok 10300, Thailand; 3 Queen Saovabha Memorial Institute, The Thai Red Cross Society, Bangkok 10330, Thailand

**Keywords:** food intake, operant conditioning, population, stray dog, trap, neuter, and release

## Abstract

**Background and Aim:**

The behavioral mechanism of stray dog occurrence is associated with domestication process. This study aimed to investigate the population and demographic relationship of stray dogs from our ecological habitat. We tested whether baited enclosure method could be used as an operant conditioning treat­ment to activate habituation behavior in stray dogs.

**Materials and Methods:**

The first investigation determined the population and demographic characteristics of stray dogs in the metropolitan city of Bangkok using the mark and recapture procedure. In the second investigation, a large cage equipped with a digital camera was used as the feeding and habituation area. Food was provided at four corners for 2 h. The approach behaviors and eating patterns were recorded during this period for 7 days.

**Results:**

The average number of stray dogs calculated within each cluster was 24 ± 6 dogs. For the natural small habitat, the density of stray dogs was 662 dogs per km^2^. This indicated that the number of dogs is underestimated using the mark and recapture procedure because of undetected puppies and shifts in the sex ratio in adult dogs. In the second investigation, we demonstrated that food was a potent positive reinforcer for stray dogs. The average onset of cage entry after offering the food was 17 min. The onset of cage entry and the reduction in the first meal duration suggested that the habituation process could be achieved within 1 week.

**Conclusion:**

The results revealed the possibility of using a large cage as a novel enclosure for food offered as the positive reinforcer for TNR program trapping procedures. We suggest that this humane trapping procedure could be used to activate habituation behavior in stray dogs.

## Introduction

Stray dogs represent a phenomenon associated with domestication as unselected dogs are left in their natural habitat with still close to humans. The pattern of human-dog bonds is complicated and plays a major role in the existence of stray dogs [1]. Owned dogs could be transformed into stray dogs after being lost, abandoned, or allowed to roam [2]. Stray dog populations are observed worldwide, even with long-term control programs by mass destruction. Stray dogs are major sources of infectious diseases for both humans and companion dogs, and dog bites and rabies infections are the most important problems related to stray dogs globally [3]. The seroprevalence of canine distemper has been equally demonstrated in both owned and stray dogs [4], and the prevalence of heartworm is high in stray dogs [5].

Similar problems related to stray dogs have been reported in Thailand [6–8]. Unfortunately, the reported population of stray dogs in Thailand, especially in Bangkok, is not completely similar to that in other regions [9, 10]. In addition, measures to control stray dog populations have not been updated. Because removal or destruction programs have been recognized as inhumane, dog control programs (DCPs) must implement other methods as core strategies to control stray dogs, including registration, neutering, and public education campaigns [11]. However, the population of stray dogs is maintained and be the major factor that interferes with DCPs [12]. This negative factor led to the idea of transforming stray dogs that are free-roaming to block dogs that are trapped, registered (microchipped), neutered, vaccinated, and released into habitats under the responsibility of the public [13]. In the metropolitan city of Bangkok, stray dogs are associated with food and humans. Temples and schools are important habitats where stray dogs aggregate and where problems related to stray dogs originate.

This study aimed to investigate the population and demographic relationship of stray dogs from our ecological habitat. We tested whether baited enclosure method could be used as an operant conditioning treatment to activate habituation behavior in stray dogs.

## Materials and Methods

### Ethical approval

The study was approved by the Animals Care and Use Committee, Faculty of Veterinary Science, Chulalongkorn University (#1531080).

### Study period and location

The demographic study of stray dogs was conducted from January to May 2015 in nine areas of Central Bangkok, including eight temples and one university. Temples were selected for this investigation to represent the small habitat typical in Bangkok for stray dogs, and a university area was selected as the large habitat where stray dogs live in Bangkok. The average temple area is 0.036 ± 0.016 km^2^ and the average university area is 0.40 km^2^. The area of both temples and universities includes mostly continuous fenced areas with some openings for humans and vehicles, where dogs can connect to the outside.

### Estimation of the stray dog population within the selected study area in Bangkok

The photo mark and recapture technique was employed to estimate the stray dog population in this study. The estimations were based on two observations per area within 1 day of observation. The estimation from each area was performed 2 times at approximately 6 months apart. On each observation day, the research team consisted of three people who observed and performed visual marking by photography, counting, and note-taking for age and sex identification. The observation trial was designed according to the preliminary trial analysis from each area. Dog identity, age, and sex were evaluated independently by the observers. Dogs were classified into four age levels: Old adults, adults, juveniles, and puppies. This classification was based on visual observations of the general appearance, determination of tooth wear if the dog could be handled, and interviews with residents who fed the dogs [14]. The first photo mark session was performed in the early morning (0600). The second or recapture session was performed along the same route in the afternoon (1700). The total population size (N) of dogs was estimated from the number of dogs in the first photo mark session (n1), the number of dogs in the recapture session (n2), and the number of marked dogs from the second session (m2) using formula 1. The lower and upper 95% confidence limits for the total population size (LCL and UCL, respectively) can be estimated with formula 2 [15].


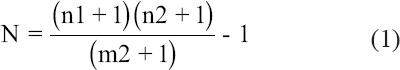



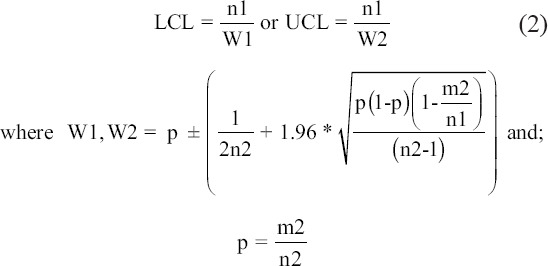


### Food-induced habituation of stray dogs

**Figure-1:**
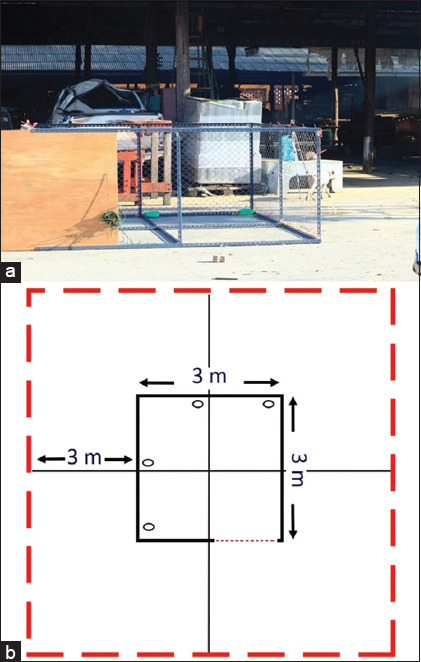
(a) Picture of the large cage used in the current investigation as the novel enclosure. (b) Diagram of the cage (small black rectangle) and the area surrounding the cage (large red rectangle) used to determine the operant conditioning. The position of the food bowls is marked with an oval shape within the cage area.

To examine the hypothesis that stray dogs could be habituated within the large cage when food is used as the reinforcer, two temples and one university were selected for this investigation according to the cluster size and the facilities. One week before the investigation, a large cage (3 × 3 m) with a 1.2 m tall wire mesh partition was installed in the study area (Figure-1a). The duration of this investigation was 7 days. The cage was equipped with a remote control door and digital camera (SJCAM SJ4000, SJCAM Limited Shenzhen Zhencheng Technology Co. Ltd., Shenzhen, China). Within the cage, food bowls were placed in four positions at the corner (Figure-1b). The area surrounding this cage (3 m from each site) was determined as the inner cage area of inspection by the dog. On each investigation day, the total observation time (120 min from 1600 to 1800) was divided into two periods: Before (10 min) and after (110 min) offering food. The observation area (approximately 10−15 m) was selected during the investigation period, where observers could clearly determine and record the information. Before offering food, the camera and the food bowls were checked. Food was offered by one observer (1610) without any interaction with the dogs. The number of dogs and the time over which they approached the inner cage area or entered the cage were counted and recorded. The onset of cage entrance started from the time of offering food until the dog entered the cage. The first meal duration was determined as the duration of the first bout of feeding, which was separated from the second bout when the dog left the feeding bowl for at least 20 min [16]. All general behavioral responses were recorded throughout the observation period, that is, urination, aggression, licking, etc.

### Statistical analysis

Data from the first investigation indicates that the estimated stray dog population is presented as the number of dogs and 95% confidence interval. The estimated number of stray dogs from two event observations was compared using the Poisson means test. Data from the second investigation are presented as the mean and standard error of measurement. Data that contained time as the factor were analyzed using repeated one-way analysis of variance (ANOVA). Data that contained two factors were analyzed using a repeated two-way ANOVA. Significance was determined at p < 0.05.

## Results

### Estimated stray dog population within the selected study area in Bangkok

A total of 230 stray dogs were estimated from the first observation (Table-1). The average number of stray dogs living in the temple area was 24 ± 6 dogs, which could be calculated based on the density of stray dogs living in small habitats of approximately 331 dogs/0.5 km^2^. Within the large habitat, the university had 41 dogs/0.4 km^2^. The sex and age structure of stray dogs from this observation revealed interesting demographic characteristics of stray dogs. First, the sex ratio of males to females in juveniles was 1:2. However, this ratio was almost 1:1 in adults and old adults. Second, the percentage of adult dogs within each observation area was 71%. Third, puppies were not observed during the sessions on the investigation day. In addition, the total number of stray dogs that were estimated from the second observation (6 months later) was 225 dogs, which was not significantly different from the value obtained from the first observation (p > 0.05).

**Table-1:** Estimated number of stray dogs by the mark-recapture technique from each observation area.

Observation	Area (km^2^)	n1	n2	m2	Estimation	95% confidence interval (LCL-UCL)
Temple						
Paiton	0.044	9	11	6	16	12–28
Intharam	0.039	12	23	10	27	22–37
Chantharam	0.02	6	8	6	8	7–9
Duangkae	0.036	12	16	12	16	15–17
Apaithayaram	0.014	7	10	5	14	10–25
Intharavihan	0.045	9	22	8	25	20–33
Mahathat	0.065	11	12	7	19	14–30
Dogmai	0.023	35	43	23	65	55–80
University	0.4	21	37	19	41	36–47
Total	0.686				230	

The number of first photo marks (n1) and second recaptures (n2) was determined within a day. The stray dog number was estimated and the confidence interval was calculated based on Equations 1 and 2, respectively, with n1 and n2 and the number of marked dogs used in the second recapture session (m2).

### Food-induced habituation in stray dogs

**Figure-2:**
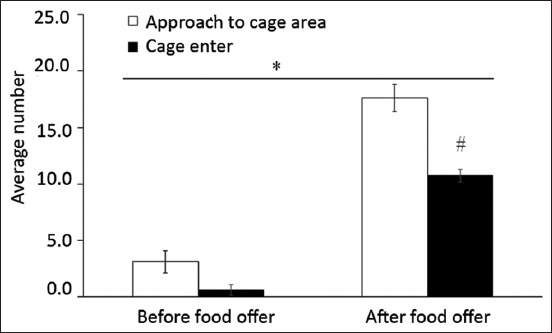
Average number of stray dogs that approached the cage area and entered the cage before and after offering food. Food was the positive reinforcer and the large cage was the novel enclosure. The major response for this operant conditioning was approaching the cage and cage entry. *Significant food effect (p < 0.05). ^#^Effect of operant conditioning (p < 0.05).

Within 10 min before offering food, the average number of dogs that approached the cage area and entered the cage was 3 ± 1.0 and 1 ± 0.5, respectively. After offering food, the average number of dogs that approached the cage area and entered the cage was 18 ± 1.2 and 11 ± 0.6, respectively. The availability of food significantly increased the attention of stay dogs in terms of approaching the cage area and entering the cage (Figure-2, p < 0.05). Before offering food, there was no difference between the number of stay dogs that approached the cage and that entered the cage (p > 0.05). After offering food, the average number of stay dogs that approached the cage was significantly higher than that entering the cage (p < 0.05).

**Figure-3:**
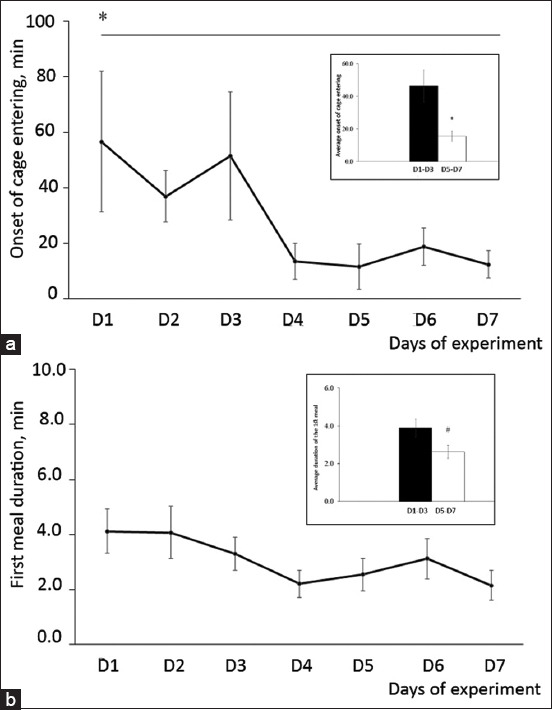
(a) Onset of cage entry and (b) first meal duration throughout the investigation days. The insets of (a) and (b) are the average onset of cage entry and first meal duration calculated from the first and last 3 days of the investigation. Effect of time *(p < 0.05), ^#^(p = 0.065).

When the onset of cage entry was considered throughout the 7 days of the food offering experiment (Figure-3a), we found that the food-induced habituation process had a significant effect on the onset of cage entry (p < 0.05), which was shorter during the last 3 days than the first 3 days (Figure-3a; p < 0.05). Although the pattern of the first meal duration across the 7 days of the food offering experiment was similar to that of the onset of cage entry (Figure-3b), the analysis revealed that this trend was not significant (p > 0.05). Similarly, the first meal duration during the last 3 days was not significantly different from that during the first 3 days (Figure-3b; p = 0.065).

## Discussion

The main objective of this investigation was to study the behavioral responses of stray dogs to novel enclosures and management strategies using food as positive reinforcement. We used food as a positive reinforcement for entrance into large cages and identified behavioral results that indicate the habituation duration of stray dogs. The results support the hypothesis that food is a powerful positive reinforcer for stray dogs that could increase desired behaviors through the learning process. After offering food within the large cage, the patterns of both onset of cage entry and meal duration were decreased, which indicated that stray dogs were habituated to the novel physical enclosure and management strategy. Moreover, demographic information on stray dogs living in the metropolitan city of Bangkok was collected, and these biological data are an important piece of information for estimating stray dog populations and further investigating control measures.

The fundamental idea of this study was to implement this technique as a humane trapping procedure for stray dogs. In general, trapping and marking are processes that require direct contact with stray dogs (i.e., restrain), which can lead to injury from dog bites. Moreover, these same processes can lead stray dogs to try and escape and hide. This investigation introduces the idea of using a feeding-induced habituation model to shape stray dogs’ behavior toward large enclosures and humans. When mastered, dogs would be easier to handle and manipulate. This technique should be implemented as part of a trap, neuter, and release (TNR) program for stray control.

Because the demographic pattern and population of stray dogs in Bangkok have not been published, we conducted our first investigation by studying the stray dog population in a selected area. Buddhist temples represent a stray dog habitat and the average area of each temple in this study was 0.036 km^2^. Based on the observation of the general behavior (urination, aggression, and licking,) one cluster of stray dogs was observed living within such a small area, with 24 ± 6 dogs/temple. Bangkok has approximately 433 registered temples. Thus, the calculated number of stray dogs living in Bangkok temples is approximately 10,392 dogs. This simple estimation is certainly lower than the actual number because it does not include other habitats (i.e., local markets, schools, etc.) where stray dogs could live. Moreover, temple clusters would provide important support for other clusters when the population within cluster increase. The size of the temple cluster of stray dogs from the current investigation was similar to the size of the university cluster, where dogs have a larger habitat [11]. When considering the average density of stray dogs, our current observation (662 dogs/km^2^) is much lower than that reported in India and Mexico [17, 18]. Both cluster size and density are fundamental factors that determine the total population of stray dogs in each city. The intervening factor is the physical separation of habitat, which plays a crucial role in providing resources (food and hidden areas) and maintaining cluster fluidity or kinetics (roaming, migration, etc.). Unfortunately, the current investigation showed that the size of stray dog clusters, which is the most important biobehavioral function, is perhaps underestimated based on two pieces of evidence. First, the male-to-female sex ratio of juveniles (1:2) was changed to 1:1 for adult stray dogs. Second, during each observation session, puppies were not included in the captured sample. Both phenomena suggested that some female stray dogs were hidden within dens, where they could safely take care of puppies. Taken together, the estimated number and demographic pattern of stray dogs from the metropolitan city of Bangkok suggest a limitation that could interfere with the accuracy of estimating the number of dogs and implementing control programs.

Habituation to novel ecological habitats is a crucial natural process of domestication [19, 20]. With this context, stray dogs are domesticated dogs that are unowned and free-living, and they learn to increase their personal distance from humans. Because habituation is one of the learning processes that can be activated in stray dogs, the current investigation aimed to shape stray dog behavior using a positive reinforcement learning process that included a novel habitat, the big cage. In principle, food has been widely used in operant conditioning for food preference assessments in dogs. However, to the best of our knowledge, this is the first investigation to use food as an operant conditioning treatment in stray dogs. We first showed that food is a strong positive reinforcer for stray dogs. To obtain food, stray dogs have to approach and enter the cage, which is a novel barrier between the dogs and food. A significant number of stray dogs approached the cage and entered the cage after offering food, which suggested the positive effect of operant conditioning. Thus, the stray dogs showed that the learning process was operant conditioning and the results indicated that habituation to the big cage could be activated within 1 week. The onset of cage entry after offering food was markedly decreased from an average 46 min during the first 3 days to 17 min during the last 3 days of the investigation. We also showed a decreasing tendency in the first meal duration, which presented a similar time point. The explanation for the longer meal duration during the first 3 days is that stray dogs engage in both alertness and eating behaviors. The shorter meal duration during the last 3 days of the investigation was only related to eating behavior and suggested that stray dogs were more habituated to our large cage. However, not all dogs that remained in the observation area came close to the cage area or entered the cage, which may imply that the degree of boldness in stray dogs within the cluster is not similar [21]. Thus, whether the stray dogs that had the highest boldness could induce the rest of the stray dogs within the cluster to accomplish this operant conditioning is an interesting question to be resolved.

Trapping and marking are important steps in TNR programs for stray dog control. Unfortunately, these are the same activities that lead stray dogs to have direct contact with humans. Moreover, unsuccessful trapping with various instruments could induce escape and hiding behavior in stray dogs. This investigation aimed to implement this operant conditioning technique for the trapping process and it not only uses food as a positive reinforcer but also requires participation from the public to complete this process. Specifically, the detailed procedure for human participation in this trapping process depends in part on government policy. In our case, participation from the public is not an impossible adjunctive process because, in reality, all stray dogs from each cluster living in the temple have caretakers. Hence, it is possible to employ one person from each nearby cluster to provide food regularly. This idea is different from the general procedure of the trapping process that uses food as bait [22]. The advantage of the whole procedure is to recruit social engagement for the stray dog problems. However, the success of the current method depends on both government policy and social acceptance. Finally, we would further hypothesize that all stray dogs within the cluster could be transformed to block dogs that could be easily handled by humans without restraint.

## Conclusion

This investigation revealed the population and demographic patterns of stray dogs in the metropolitan city of Bangkok. The collected information suggested that puppies and some adult females may not be included in the population estimates, which lead to inaccurate estimates. This investigation indicates that habituation behavior in stray dogs could be reactivated using an operant conditioning technique. Finally, within each stray dog cluster, differences occur in personal temperament and boldness, which may contribute to the habituation process. Finally, the information from this investigation implicates the human management procedure that includes the participation of public and government in taking care of stray dogs in the city.

## Authors’ Contributions

SS, VC, PT, KN, and ST: Contributed to the conception and designed the study. ST: Contributed materials and analysis tools. SS, VC, PT, KN, and ST: Performed the animal experiments. ST: Analyzed the data and performed the statistics. NC and ST: Wrote and revised the manuscript. All authors contributed to the drafting and revision of the manuscript. All authors have read and approved the final manuscript.
